# Attentional Phenotypes for the Analysis of Higher Mental Function

**DOI:** 10.1100/tsw.2002.93

**Published:** 2002-01-24

**Authors:** John Fossella, Michael I. Posner, Jin Fan, James M. Swanson, Donald W. Pfaff

**Affiliations:** Sackler Institute for Developmental Psychobiology, Department of Psychiatry, Weill Medical College of Cornell University, 1300 York Avenue, New York, NY, 10021, USA; Child Behavior Center, University of California at Irvine, USA; Laboratory of Neurobiology and Behavior, Rockefeller University, USA

**Keywords:** attention, cognition, candidate genes, imaging

## Abstract

We outline a strategy to relate normal cognitive processes to candidate genes. First, brain imaging is used to specify a cognitive process “attention” in terms of the neural networks involved. Next, evidence is presented showing that the operation of each network involves a dominant neuromodulator. Then we discuss development of a task designed to measure the efficiency of each network in normal individuals and consider evidence on the independence, reliability, and heritability of the networks. DNA from cheek swabs of subjects who performed the task are then used to examine candidate polymorphisms in genes related to the transmitters. We then examine the ability of these candidate alleles to predict the efficiency of relevant networks. This process has demonstrated that candidate genes are related to specific networks of attention to a greater degree than to overall performance as measured by reaction time and accuracy. These findings require replication and possible extension to other cognitive processes.

## INTRODUCTION

There is considerable interest in being able to specify the phenotypes needed for genetic analysis of higher mental function. Most of the discussion has involved psychiatric and neurological disorders that influence cognitive processes; however, it would be clearly advantageous to understand the genetic basis of normal cognition because it might allow us to understand individual differences among normal people as well as illuminating various disorders.

## FUNCTIONAL ANATOMY

Functional neuroimaging has allowed many cognitive tasks to be analyzed in terms of the brain areas they activate. Studies of attention have been among the most often examined in this way. Imaging data have supported the presence of three networks related to different aspects of attention. These networks carry out the functions of alerting, orienting, and executive control[1,2].

Alerting is defined as achieving and maintaining a state of high sensitivity to incoming stimuli; orienting is the selection of information from sensory input; and executive control is defined as involving the mechanisms for resolving conflict among thoughts, feelings, and responses. The alerting system has been associated with the frontal and parietal regions of the right hemisphere. A particularly effective way to vary alertness has been to use warning signals prior to targets. The influence of warning signals on the level of alertness is thought to be due to modulation of neural activity by the norepinepherine (NE) system[3].

Orienting involves aligning attention with a source of sensory signals. This may be overt, as in eye movements, or may occur covertly without any movement. The orienting system for visual events has been associated with posterior brain areas including the superior parietal lobe and temporal parietal junction and, in addition, the frontal eye fields. Orienting can be manipulated by presenting a cue indicating where in space a person should attend, thereby directing attention to the cued location[4]. Event-related functional magnetic resonance imaging studies have suggested that the superior parietal lobe is associated with orienting following the presentation of a cue[5]. The superior parietal lobe in humans is closely related to the lateral intraparietal area in monkeys, which is known to produce eye movements[6]. When a target occurs at an uncued location and attention has to be disengaged and moved to a new location, there is activity in the temporal parietal junction[5]. Lesions of the parietal lobe and superior temporal lobe have been consistently related to difficulties in orienting[7].

Executive control of attention is often studied by tasks that involve conflict, such as various versions of the Stroop task. In the Stroop task, subjects must respond to the color of ink (e.g., red) while ignoring the color word name (e.g., blue). Resolving conflict in the Stroop task activates midline frontal areas (anterior cingulate) and lateral prefrontal cortex[8,9]. There is evidence for the activation of this network in tasks involving conflict between a central target and surrounding flankers that may be congruent or incongruent with the target[10,11,12]. Experimental tasks may provide a means of fractionating the functional contributions of different areas within the executive attention network[9,11,12].

## CHEMICAL MODULATION

It is very important to be able to link the neurosystem results—those that suggest specific brain regions related to attention—with cellular and synaptic studies. One strategy for doing so is to study the pharmacology of each of the attention networks. To carry out these tests it is useful to use alert monkeys who are able to perform relevant attentional tasks[3,13].

A series of pharmacological studies with alert monkeys has related the attentional networks we have discussed with specific chemical neuromodulators[3,13]. Alerting is thought to involve the cortical distribution of the brain's NE system arising from neurons with cell bodies in the locus coeruleus of the midbrain. Drugs like clonidine and guanfacine act to block NE and reduce or eliminate the normal effect of warning signals on reaction time, but have no influence on orienting to the target location[3].

Cholinergic systems arising in the basal forebrain play a critical role in orienting. Lesions of the basal forebrain in monkeys interfere with orienting attention[14]. It does not appear, however, that the site of this effect is in the basal forebrain; instead, it appears to involve the superior parietal lobe. Injections of scopolamine directly into the lateral intraparietal area of monkeys, a brain area containing cells that are influenced by cues about spatial location, have been shown to have a large effect on the ability to shift attention to a target[13]. Systemic injections of scopolamine have a smaller effect on covert orienting of attention than do local injections in the parietal area. Cholinergic drugs do not affect the ability of a warning signal to improve performance; thus, there is a double dissociation that relates NE to the alerting network and acetylcholine to the orienting network. These observations in the monkey have also been confirmed by similar studies in the rat[15]. Of special significance in the rat are comparisons of the cholinergic and dopaminergic mechanisms that have shown that only the former influence the orienting response[15,16].

The executive network is thought to involve the anterior cingulate and lateral frontal cortex modulated by a dopamine (DA) system with cell bodies arising in the ventral tegmental region. The cingulate in particular shows expression of all of the DA receptors[17,18]. We regard DA as the dominant neuromodulator for the executive network.

## DESIGN OF PHENOTYPES

To study individual differences in these networks we have developed an attention network test (ANT) that examines the efficiency of the three brain networks we have discussed above[19]. We used differences in reaction times (RT) derived from the task shown in Fig. 1. Subtracting RTs obtained in the double-cue condition from RT in the no-cue condition gives a measure of alerting due to a warning signal. Subtracting RTs to targets at the cued location from trials using a central cue gives a measure of orienting. Subtracting congruent from incongruent target trials provides a measure of conflict. The data provide three numbers that represent the skill of each individual in the alerting, orienting, and executive networks. In a sample of 40 normal persons we found these numbers to be reliable over two successive presentations. In addition, we found no correlation among the three network numbers. An analysis of the reaction times found in this task shows large main effects for cueing and for the type of target, but only two small but significant interactions[19]. They both involve very small reductions in conflict when either no warning cue is provided or when the cue is at the location of the target. The latter interaction is to be expected because the effective eccentricity of flankers is increased when attention is specifically on the central arrow. The first finding appears to arise because with no warning the subject is generally slow and some conflict resolution may occur during the longer overall RT. This effect also appears to be responsible for a small but significant negative correlation between alerting and conflict ($r = -.18$, $p < .01$) when we examine a larger sample of more than 200 people who have taken the ANT.

The ability to measure differences in attention among adults raises the question of the degree to which attention is heritable. In order to deal with this issue we used the ANT to study 26 pairs of monozygotic and 26 pairs of dizygotic same-sex twins[20]. We found strong correlations between the monozygotic twins for both the executive ($r = 0.73$) and alerting networks ($r = 0.46$). For the alerting network we found a similar although somewhat smaller correlation among the dizygotic twins ($r = 0.38$); however, for the executive network the dizygotic twins were only slightly correlated (0.28) yielding a high heritability of 0.89. Because of the small sample size, the estimates of heritability are not very precise. Nonetheless these data support a role for genes in accounting for variations in efficiency in the executive and possibly in the alerting networks. Our current studies of normal children suggest that the conflict and alerting networks continue to develop until late childhood or adolescence, 10-year-old children having somewhat more difficulty resolving conflict and less ability to maintain alertness than adults do in the same version of the ANT[21].

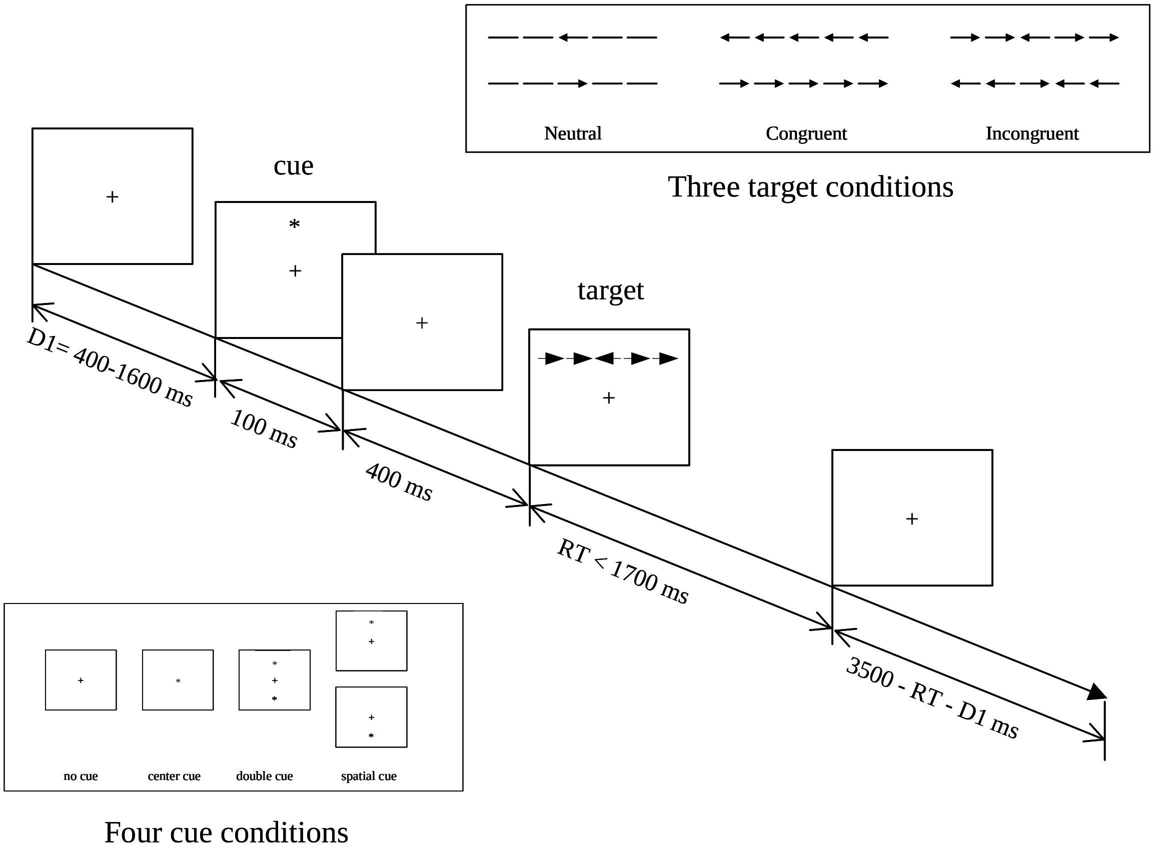

**FIGURE 1.** Attention network task. The target is an array of five arrows (see three target conditions at top of figure) presented above or below fixation, to which the person responds by pressing a key in the direction of the central arrow as quickly as possible. Prior to the target, cues are introduced to produce alerting and orienting to the target location (see cue conditions box).

## CANDIDATE GENES

We have used the relationship of the alerting and executive networks with the neuromodulators NE and DA as a way of choosing candidate genes that might relate to the efficiency of these networks. To examine these relationships we have tested more than 200 persons in the ANT and have obtained DNA from buccal swabs to examine frequent polymorphisms in genes related to their respective neuromodulators (see Fig. 2). We plan to report our detailed genetic methods and results in a separate paper[22]; however, we have obtained preliminary evidence of the association of several of the most frequently studied DA- and NE-related genes. These genes appear to be associated with individual networks and not to overall reaction time or accuracy. This result provides some support for the utility of the attentional phenotypes we have defined and suggests they may be at the appropriate level to be useful in the study of molecular genetics.

To illustrate the nature of our findings, consider the alleles of the dopamine D4 receptor (DRD4) gene widely reported to be associated with attention deficit disorder[23]. Previously, we were surprised to find that children diagnosed with attention deficit hyperactivity disorder (ADHD) who have the 7-repeat allele did not have deficits in overall RT or in conflict as measured by the color-word Stroop task[24]; however, new data (Fig. 3) provide a more complete picture. We find that among normal persons, the 4-repeat allele of this gene does have a small but significant effect specifically on the conflict and alerting networks. Indeed the 4,7 combination shows the greatest difficulty with the conflict network. The presence of the 4-repeat allele appears

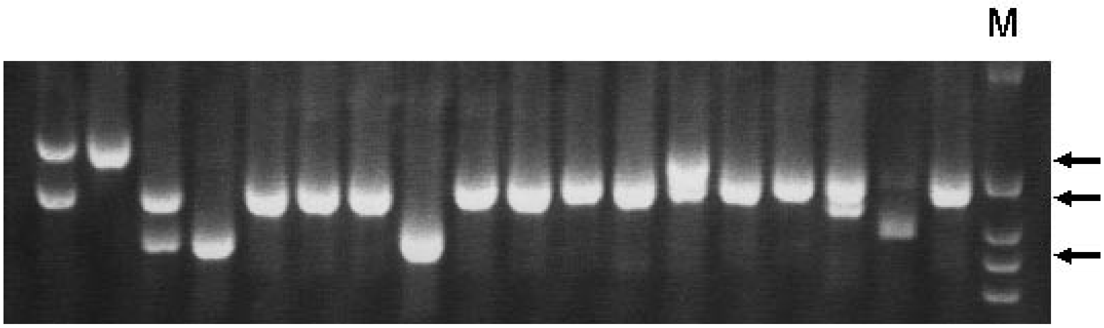

**FIGURE 2.** Gel electrophoregram showing results of PCR amplification of the DRD4 exon III VNTR from genomic DNA obtained from buccal cells from test subjects. Uppermost band represents 7-repeat allele of the VNTR; the middle band represents a 4-repeat polymorphism; and the lower band represents a 2-repeat allele. The last lane contains molecular weight standards.

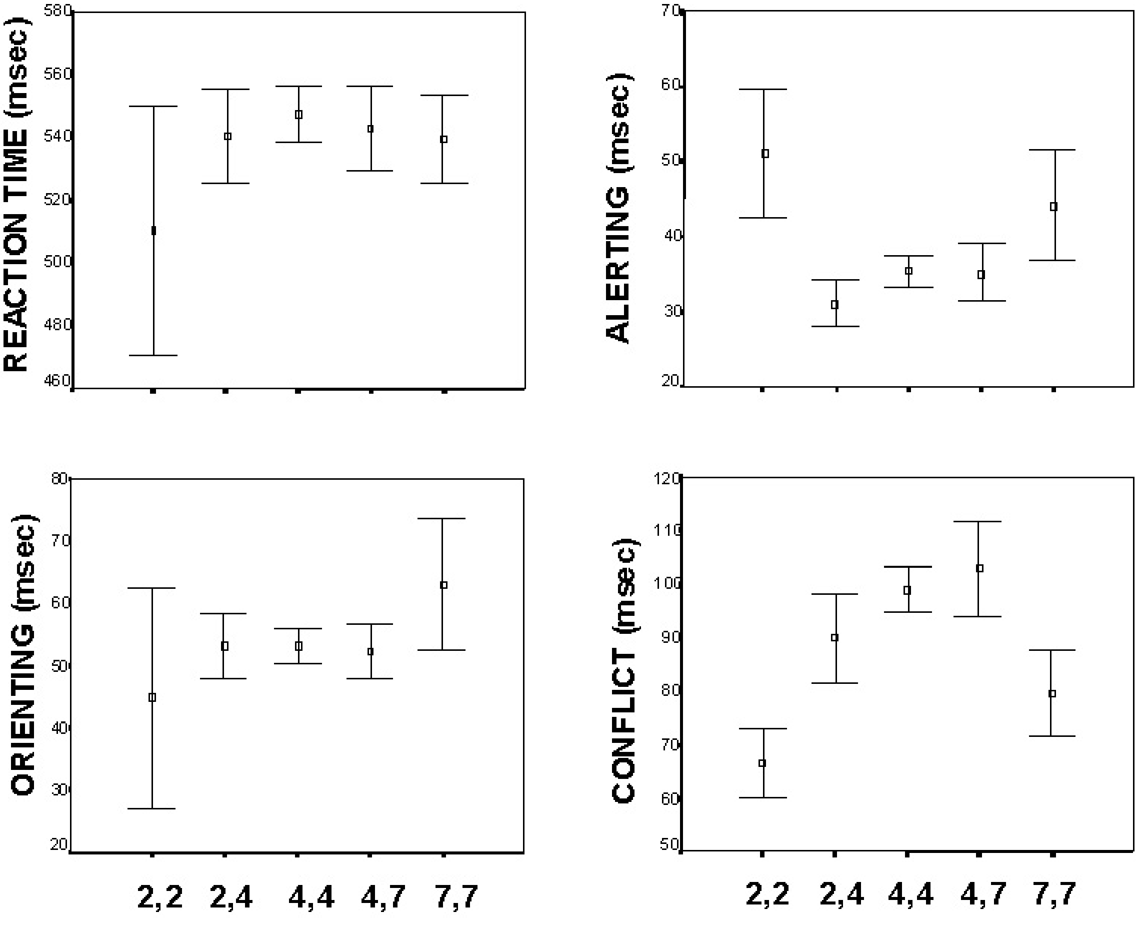

**FIGURE 3**. Plots showing the distribution of attention network performance scores vs. DRD4 genotypes. The three most frequent alleles of a 48-bp repeat in exon III can be grouped into five genotypic classes: (2-repeat homozygotes; 2,4 heterozygotes; 4-repeat homozygotes; 4,7 heterozygotes; and 7-repeat homozygotes). The bars represent mean ±SE for each genotypic class. The number of subjects for each genotypic class is 2,2 (n = 5); 2,4 (n = 28); 4,4 (n = 121); 4,7 (n = 37); and 7,7 (n = 9).

to be associated with more difficulty in resolving conflict and to a smaller degree with a reduced alerting effect, but has no association with overall RT or accuracy. The association of several polymorphisms in the DRD4 gene with conflict among normal subjects[21] provides additional impetus for consideration of the role of this gene in disorders such as ADHD. The data from these candidate gene studies are still incomplete. At this time we are continuing the search both in normal subjects and in appropriate patient populations.

## CONCLUSION

The networks of attention are probably the best studied of all cognitive processes, but the strategy we have described of using neuroimaging and psychopharmacology to develop candidate genes could be widely applied to other cognitive functions. Our results to date suggest that phenotypes defined on the basis of the efficiency of specific neural networks, rather than general features of overall performance such as RT or accuracy, may be more closely associated with specific genes. The strategy we have used invites replication and extension to other cognitive processes.

## ACKNOWLEDGMENTS

This research was supported in part by a 21st Century Award from the James S. McDonnell Foundation and by NSF Grant BCS 9907831. The authors are grateful to other members of the Sackler Institute for their support and help.

## BIOSKETCHES

**John Fossella** is a Research Fellow at the Sackler Institute. He received his Ph.D. in molecular biology from Princeton and is studying the role of genes in normal and abnormal cognitive processes such as attention and attention deficit hyperactivity disorder.

**Michael Posner** is Professor and Director of the Sackler Institute. He conducts research on human brain development with special emphasis on development of attentional networks.

**Jin Fan** is a Research Fellow at the Sackler Institute. He received his Ph.D. from New York University and has been studying the neural basis of attentional networks at the Institute.

**James Swanson** is Director of the Child Development Center, U.C. Irvine and Senior Researcher at the Sackler Institute, Dr. Swanson has worked on all aspects of attention deficit hyperactivity disorder (ADHD) and is currently studying the molecular genetics of ADHD particularly in reference to the dopamine D4 receptor gene.

**Donald Pfaff** heads the laboratory of Neurobiology and Behavior at Rockefeller University which studies hormonal effects on gene experession that control arousal behavior. He is currently working jointly with the Sackler Institute on the genetics of attention.
